# A Novel Hybrid Approach for Drowsiness Detection Using EEG Scalograms to Overcome Inter-Subject Variability

**DOI:** 10.3390/s25175530

**Published:** 2025-09-05

**Authors:** Aymen Zayed, Nidhameddine Belhadj, Khaled Ben Khalifa, Carlos Valderrama, Mohamed Hedi Bedoui

**Affiliations:** 1 Service d’électronique et de Microélectronique, University of Mons, 7000 Mons, Belgium; carlos.valderrama@umons.ac.be; 2 Research Laboratory of Technology and Medical Imaging-LTIM-LR12ES06, Faculty of Medicine of Monastir, Monastir 5000, Tunisia; khaled.benkhalifa@issatso.rnu.tn (K.B.K.); medhedi.bedoui@fmm.rnu.tn (M.H.B.); 3 National Engineering School of Sousse, University of Sousse, Sousse 4054, Tunisia; 4 Laboratory of Electronics and Microelectronics, Faculty of Sciences of Monastir, Monastir 5019, Tunisia; nidhameddine.belhadj@fsm.rnu.tn; 5 Higher Institute of Applied Science and Technology of Sousse, Sousse 4003, Tunisia

**Keywords:** drowsiness, scalogram, EEG, deep learning, CNN, transfer learning

## Abstract

Drowsiness constitutes a significant risk factor in diverse occupational settings, including healthcare, industry, construction, and transportation, contributing to accidents, injuries, and fatalities. Electroencephalography (EEG) signals, offering direct measurements of brain activity, have emerged as a promising modality for drowsiness detection. However, the inherent non-stationary nature of EEG signals, coupled with substantial inter-subject variability, presents considerable challenges for reliable drowsiness detection. To address these challenges, this paper proposes a hybrid approach combining convolutional neural networks (CNNs), which excel at feature extraction, and support vector machines (SVMs) for drowsiness detection. The framework consists of two modules: a CNN for feature extraction from EEG scalograms generated by the Continuous Wavelet Transform (CWT), and an SVM for classification. The proposed approach is compared with 1D CNNs (using raw EEG signals) and transfer learning models such as VGG16 and ResNet50 to identify the most effective method for minimizing inter-subject variability and improving detection accuracy. Experimental evaluations, conducted on the publicly available DROZY EEG dataset, show that the CNN-SVM model, utilizing 2D scalograms, achieves an accuracy of 98.33%, outperforming both 1D CNNs and transfer learning models. These findings highlight the effectiveness of the hybrid CNN-SVM approach for robust and accurate drowsiness detection using EEG, offering significant potential for enhancing safety in high-risk work environments.

## 1. Introduction

Vigilance is defined as the state of optimal reactivity of the organism to external or internal stimuli, encompassing the ability to maintain attention and respond appropriately to various situations over an extended period [1]. This alertness is essential for cognitive performance, safety, and productivity, and it is influenced by internal factors such as fatigue and external factors such as the environment. Reduced alertness, often caused by insufficient sleep, poor sleep quality, circadian rhythm disruptions, or medical conditions [2], impairs attention and increases the risk of errors, slower reactions, and accidents [3].

Human vigilance is primarily divided into four states: deep sleep [4]; light sleep [5]; active wakefulness [6]; and passive wakefulness, also known as drowsiness [7]. Drowsiness is characterized by a decrease in alertness and an increased tendency to relax. Although this state typically lasts only a few minutes, its consequences can be disastrous in many professional sectors, such as medicine, construction, industry, and transportation. Drowsiness can lead to serious accidents, which is why it is so important to detect and prevent it effectively.

Drowsiness detection [8] relies on various methods, each with its advantages and limitations. Traditional methods include self-reported questionnaires [9], behavioral observation, and the use of cameras to analyze facial expressions or eye movements [10]. While these methods are useful, they often lack precision and objectivity. For example, facial expression-based methods analyze facial features such as eyelid opening, yawning, and eye movements to detect drowsiness. These methods can be affected by lighting conditions, visual obstructions, or distractions, and may not detect early signs of drowsiness before they become apparent.

Analysis of EEG signals [11], on the other hand, offers an advanced and effective method of detecting drowsiness. EEG measures the brain’s electrical activity via electrodes placed on the scalp, providing a direct perspective on the brain’s state. This method enables the identification of subtle drowsiness-related changes in brain activity through EEG, often before visible signs emerge [12,13]. EEG is particularly effective for early drowsiness detection as it captures neural changes that typically precede observable symptoms. Moreover, EEG provides objective and quantifiable data, unlike observation-based or self-reported methods. EEG-based systems can then monitor alertness in real time, enabling rapid intervention, which is crucial in critical environments such as driving or operating machinery.

EEG signals, while highly effective for detecting drowsiness, are extremely sensitive to artifacts caused by muscle movements and power line interferences. This sensitivity necessitates a rigorous artifact elimination process to ensure data quality. The size of EEG segments is also a critical parameter for accurate and reliable detection, as it directly impacts the effectiveness of feature extraction.

The frequency characteristics of EEG signals are valuable markers for the detection of drowsiness [14]. However, the large variability between subjects poses problems, leading to substantial differences in indicators of drowsiness between individuals. Temporal characteristics further contribute to understanding vigilance states, making the integration of time-frequency features particularly advantageous. Time-frequency representations have the potential to mitigate the impact of inter-subject variability, thereby enhancing detection accuracy. In this context, the Continuous Wavelet Transform represents a highly effective method for generating time-frequency representations of EEG signals, producing scalograms that are particularly useful for advanced analysis and classification [15].

With the advent of artificial intelligence (AI) [16], numerous AI-based drowsiness detection approaches using EEG signals have emerged. Several studies focus on classical machine learning (ML) tools [17], such as SVMs, due to their ability to handle the non-linearity of EEG data and their classification performance. However, classical ML tools are limited in their capacity to manage high-dimensional data and extract features.

A growing body of research is exploring the possibility of using deep learning (DL) tools for drowsiness detection from EEG signals [18], particularly convolutional neural networks [19]. Originally designed to process images, CNNs have demonstrated exceptional performance due to their ability to extract complex and relevant features from visual data.

SVMs excel in classification and handling EEG data non-linearity, while CNNs are superior in feature extraction and processing high-dimensional data like EEG scalograms [20]. A hybrid CNN-SVM approach, combining both strengths, would make the detection of drowsiness from EEG signals particularly effective.

This study proposes a generalized approach for EEG-based drowsiness detection that addresses inter-subject variability thanks to the rich time-frequency information provided by scalograms and the effectiveness of the CNN-SVM combination. By taking advantage of the richness of the data provided by scalograms, the computational efficiency of CNN-based feature extraction, and the classification capability of SVMs, this approach aims to significantly improve the accuracy and reliability of sleepiness detection, even in the presence of significant individual differences between subjects.

The key contributions of this work are as follows:

- We propose a hybrid CNN-SVM model that combines a CNN for automatic feature extraction with an SVM for robust classification, improving drowsiness detection accuracy.
- We utilize EEG scalograms to extract meaningful time-frequency features, preserving both temporal and frequency information for enhanced detection performance.
- We mitigate inter-subject variability by designing a model that learns generalized patterns across different individuals, ensuring consistent and reliable drowsiness detection.

This paper is structured as follows: Section 2 provides an overview of the latest developments related to this research. In Section 3, we develop the proposed methodology, providing a detailed explanation. Section 4 presents the experimental results on which we build a detailed discussion. Finally, Section 5 provides a concise conclusion and explores future orientations for this study.

## 2. Related Works

Drowsiness detection has gained significant attention in recent years due to its critical role in ensuring safety, especially in transport and healthcare. Among the various proposed techniques, EEG signal analysis is distinguished by its direct correlation with brain activity. EEG-based drowsiness detection analyzes complex neuronal oscillation patterns to distinguish between wakefulness and drowsiness. To enhance the accuracy and reliability of such systems, researchers are increasingly adopting ML and DL techniques. The capacity of these models to learn complex structures and data mappings offers a promising solution to the challenges associated with EEG signal variability across individuals and conditions. Building on these advancements, several studies have explored innovative approaches to further refine EEG-based drowsiness detection systems.

Abidi et al. in [21] introduced a novel approach for drowsiness detection using 10-s EEG segments, employing the Time-Quasi-Tangent Wavelet Transform (TQWT) to extract key EEG sub-bands—alpha and theta—along with nine temporal features. Subsequently, Kernel Principal Component Analysis (K-PCA) was employed to reduce the dimensionality of these features while preserving system performance. To detect reduced vigilance, they implemented two machine learning algorithms: KNN and the SVM. Tests on laboratory participants showed that the SVM classifier achieved around 94% accuracy in intra-subject mode and 83% in inter-subject mode.

Hui Wang et al. [22] proposed an innovative approach for drowsiness detection by processing EEG signals recorded from 32 electrodes. They used two filters to eliminate interferences in EEG signals: a high-pass filter at 0.5 Hz and a low-pass filter at 30 Hz. The cleaned EEG signals were then segmented into 5-s intervals. Each segment was transformed into a time-frequency representation (spectrogram), using the Fourier transform to extract spectral features. For detecting the subjects’ states (alert or drowsy), an SVM coupled with features selection using genetic algorithms (GA) was employed. This approach achieved an accuracy of 85% in drowsiness detection.

Jian Cui et al. [23] proposed a CNN for drowsiness detection using a single EEG electrode (the Oz channel), demonstrating its ability to classify wakefulness and drowsiness states. EEG signals from 11 subjects were filtered using two finite impulse response (FIR) filters, a 1 Hz high-pass filter, and a 50 Hz low-pass filter before being segmented into 3-s intervals. These segments were input into the CNN for feature extraction, followed by classification using a dense softmax layer. The method achieved a classification accuracy of 73.22% in the inter-subject mode.

Hanan Bin Obaidan et al. [24] introduced a deep multi-scale convolutional neural network (EEG DMNet) 1D for drowsiness detection using EEG signals. Their approach utilized the frequency characteristics of pre-processed EEG signals from 17 electrodes (FT7, FT8, T7, T8, TP7, TP8, CP1, CP2, P1, P2, PO3, POZ, PO4, O1, Oz, and O2), segmented into 8-s intervals, and fed into the EEG DMNet model. This method achieved an accuracy of 95.35%, a sensitivity of 95.35%, and an F1 score of 95.33%.

Dongrui Gao et al. [25] present an innovative detecting drowsiness method using EEG signals, log-Mel spectrograms, and recurrent convolutional neural networks (CRNNs). EEG signals are first transformed into log-Mel spectrograms, which provide a detailed frequency-domain representation. These spectrograms are then input into the CRNN, combining the local feature extraction capabilities of CNNs and the temporal modeling strengths of RNNs for temporal dependencies. Experimental results demonstrate that this approach achieves a classification accuracy of more than 90% in inter-subject mode.

M et al. [26] propose a deep neural network hybrid approach for recognizing states of alertness from EEG signals to improve road safety by detecting drowsiness early. Their method combines 1D CNNs and recurrent neural networks (RNNs) to exploit the unique characteristics of EEG signals. The process begins with the pre-processing of EEG data to eliminate noise and artifacts. Following this, CNNs are employed to extract relevant local features from signal segments, capturing crucial spatial information. Long short-term memory networks (LSTMs), a variant of RNNs, are then used to model the temporal dependencies between these extracted features. This combination enables the model to identify complex patterns associated with drowsiness in EEG data. The output from the RNN layers is subsequently processed by fully connected layers to classify states of vigilance and fatigue. Experimental results demonstrate that this hybrid approach achieves a classification accuracy of 82.73%.

Luis Guarda et al. [27] introduce a novel model based on the capsule neural network (CapsNet) for detecting drowsiness from EEG signals. This model is notable for its ability to capture hierarchical spatial relationships between EEG signal characteristics, offering an advantage over conventional CNNs. The methodology begins with the pre-processing of EEG signals from five electrodes (Fz, Cz, C3, C4, and Pz) by using a band-pass filter to isolate relevant frequencies, typically ranging from 0.5 to 50 Hz. The filtered signals are then transformed into spectrograms to analyze the data in the frequency domain. The model architecture employs CapsNet to capture intricate relationships and fine details within the data. Experimental results demonstrate that this approach achieves a sleepiness detection accuracy of 86.44% in the inter-subject mode, showcasing its effectiveness in identifying drowsiness.

In [28], Amal Boudaya et al. propose a method for detecting hypovigilance using EEG signals and 1D CNNs. EEG data are collected from 32 electrodes arranged according to the 10–20 system. The signal preprocessing includes the application of a bandpass filter to isolate relevant frequencies (0.5–50 Hz) and a notch filter to eliminate 50 Hz interference. EEG segments are extracted in 5-s slices and transformed into spectrograms, which provide detailed frequency representations of the signals. These spectrograms serve as inputs to the CNN model, which features convolutional layers for feature extraction, pooling layers for dimensionality reduction, and fully connected layers for final classification. The results demonstrate that the model achieves a hypovigilance classification accuracy of over 93.94%. In [29], S. Pérez-Velasco et al. proposed a deep learning model (EEGSym) to reduce inter-subject variability in EEG signals for motor imagery-based BCI applications. The architecture combines Inception and Residual blocks for advanced spatio-temporal analysis, along with standard convolution, pooling, and flatten layers. EEG segments of 3 seconds were used, and the model was evaluated using both 8 and 16 electrodes. The 8-electrode configuration included F3, C3, P3, Cz, Pz, F4, C4, and P4, while the 16-electrode setup added F7, T7, P7, O1, F8, T8, P8, and O2. The model achieved a maximum accuracy of 88.4% with 8 electrodes and 90.2% with 16. Although this method presents a valuable approach for addressing inter-subject variability, it is tailored for motor imagery tasks rather than drowsiness detection. Additionally, its high architectural complexity, short segment length (3 s), and large number of required electrodes limit its suitability for embedded real-time drowsiness detection systems, where simplicity, efficiency, and minimal electrode usage are crucial.

In [30], k Vo et al. proposed an approach combining graphical models with generative adversarial networks to model EEG signals by disentangling latent space representations, enabling the separation of interpretable temporal and spatial components and generation of realistic synthetic EEG data. While this method shows promise for handling inter-subject variability, our study focuses instead on time-frequency representations and CNN–SVM classification rather than generative modeling.

In the work [31], J.-H. Kim et al. propose a deep learning model designed to minimize inter-subject variability in emotion recognition by leveraging temporal resolution. The model was evaluated on two datasets, an in-vehicle whole-brain EEG dataset and a prefrontal EEG dataset, achieving accuracies of 80.48% and 86.3%, respectively. While this approach demonstrates strong performance in reducing inter-subject variability, it remains relatively complex for embedded implementation and requires a high number of electrodes (32), making it less suitable for resource-constrained systems such as real-time drowsiness detection devices.

The reviewed works mainly focus on drowsiness detection using machine learning and deep learning techniques, with a particular emphasis on CNN-based models. Most studies apply filtering techniques during preprocessing, generally targeting the 0.1–50 Hz frequency range. However, a major limitation lies in their exclusive focus on frequency characteristics, often neglecting the temporal dynamics of EEG signals, which are crucial for tracking the gradual onset of drowsiness. In addition, while many approaches employ 1D CNNs, they may not fully exploit the potential of CNN architectures, which tend to perform better with 2D data. This suggests that the opportunity to capture the rich spatial and temporal information embedded in 2D EEG representations has often been missed.

The practicality of these methods is further limited by the frequent use of a large number of electrodes, which increases hardware complexity and reduces suitability for real-world applications. Another challenge is the use of short analysis segments that may lack sufficient information to reliably detect early signs of drowsiness. Moreover, inter-subject variability remains a critical obstacle, often reducing detection accuracy when systems are applied to new users without calibration.

The proposed approach addresses these limitations through several complementary innovations that go beyond the simple fusion of CNN and SVM. First, scalograms generated by the Continuous Wavelet Transform (CWT) are used to combine both time and frequency information, capturing subtle spectral changes over time that are strongly linked to drowsiness. This contrasts with prior studies that limit analysis to a single domain. Second, a lightweight yet effective 2D CNN architecture is employed to extract discriminative features from EEG scalograms, enabling higher representational power while keeping computational requirements low an essential condition for deployment in real-time embedded systems such as FPGAs. Third, replacing the final softmax layer with an SVM using an RBF kernel improves binary classification by handling the non-linear and high-dimensional nature of EEG patterns associated with drowsiness.

Another key contribution is the explicit consideration of inter-subject variability, addressed by using standardized time–frequency features to improve generalization across subjects without individual calibration. Finally, the system achieves high accuracy using only two EEG derivations, unlike many existing methods that rely on numerous electrodes, making it more practical, energy-efficient, and adaptable to daily-life applications where wearable or portable drowsiness monitoring is required.

## 3. Methodology

**Figure 1:**
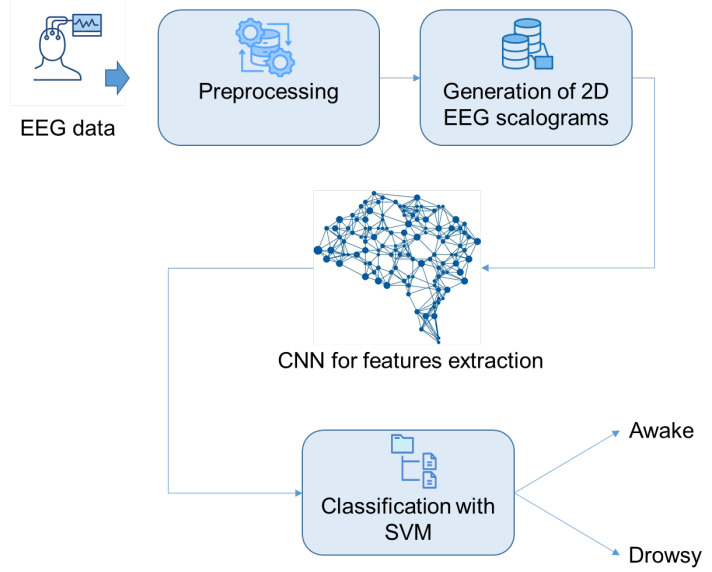
The suggested drowsiness detection workflow.

**Figure 2:**
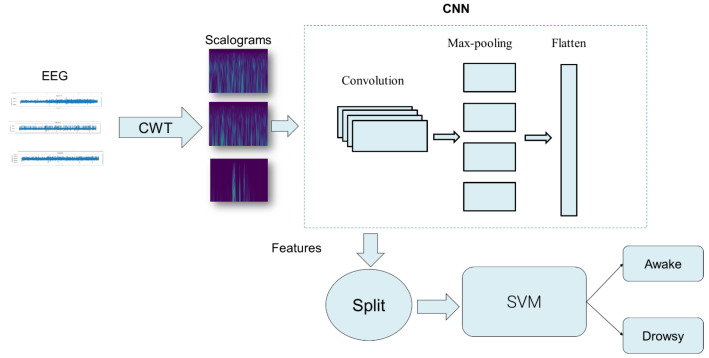
Comprehensive overview of features extraction and classification of the proposed method.

This study focuses on the detection of drowsiness using EEG signals. To achieve this goal, our approach begins by applying a rigorous pipeline to remove unwanted artifacts from EEG segments. Next, these segments are converted into scalograms using CWT. The resulting scalograms, which are rich in time-frequency information, are used as input to a 2D CNN. The CNN is responsible for automatically extracting distinctive features from the scalograms. Finally, an SVM classifier is used to detect drowsiness states. Figure 1 and Figure 2 illustrate in detail the various stages of the proposed method for detecting drowsiness.

### 3.1. DROZY Database

The selection of an appropriate database is a crucial factor in the development of robust drowsiness detection systems. While numerous publicly available datasets predominantly focus on sleep onset, this study targets the detection of drowsiness as a distinct state. Accordingly, the ULg Multimodality Drowsiness Database (DROZY) [32] was utilized. DROZY includes high-quality recordings from five EEG channels (Fz, Cz, C3, C4, and Pz) stored in EDF format and sampled at 512 Hz. This dataset is specifically designed to investigate vigilance states in 14 healthy participants, all screened to exclude drug use, alcohol consumption, and sleep disorders.

**Figure 3:**
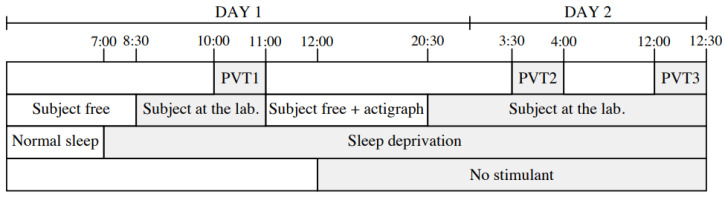
Protocol for collecting data per subject over 2 days, organized by activity and PVT (Psychomotor Vigilance Task) sessions [32].

**Figure 4:**
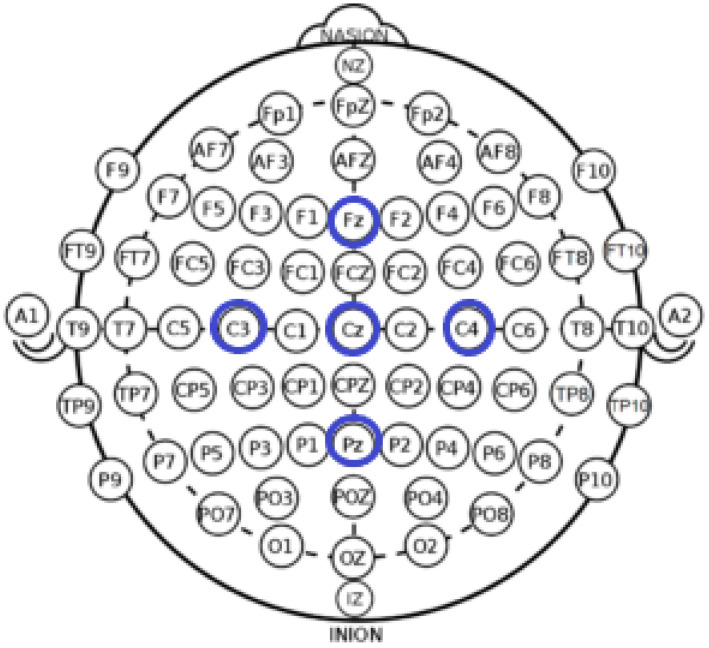
Distribution and Labeling of EEG Electrodes (10–20 Standard) with Highlighted Positions for the DROZY Database (Fz, Cz, C3, C4, Pz in Blue Circles) [33].

The data collection protocol required participants to perform a Psychomotor Vigilance Task (PVT) three times across two consecutive days, amounting to 28.30 hours of sustained wakefulness. For each PVT session, 10 minutes of EEG signal recordings are collected per subject. These sessions were strategically distributed across morning, afternoon, and nighttime periods to capture variations in vigilance levels. The experimental protocol is comprehensively outlined in Figure 3. Electrodes placement adhered to the internationally standardized 10–20 system [33], as depicted in Figure 4, ensuring accurate and reliable EEG recordings. Following each PVT session, participants were asked to report their subjective vigilance levels using the Karolinska Sleepiness Scale (KSS) [34]. The KSS, a nine-point scale widely used to assess sleepiness, provides a granular representation of vigilance states.

**Figure 5:**
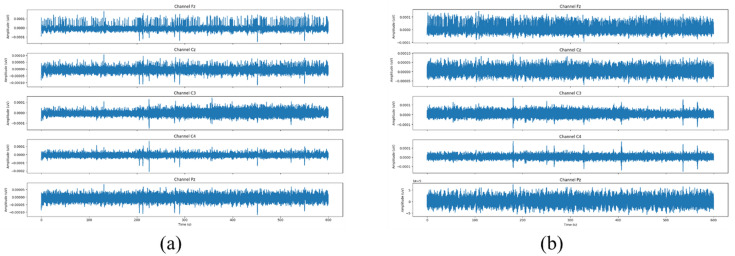
EEG signals for an alert (**a**) and a drowsy (**b**) individual [33].

In this study, we just aim to detect drowsiness, so we do not need to differentiate specific intermediate vigilance levels. Therefore, levels 0, 1, 2, and 3 are grouped as level 0 (alert), while levels 4, 5, 6, 7, 8, and 9 are classified as level 1 (drowsy). Figure 5 displays the EEG signals for both, an alert and a drowsy state individual.

### 3.2. Preprocessing

For drowsiness detection using EEG signals, the preprocessing phase is crucial [35], as it significantly affects both the performance and the accuracy of detection. Indeed, EEG signals are particularly sensitive to artifacts [36], which can be either physiological or non-physiological. To minimize the effect of these artifacts, several removal techniques have been developed. Many studies have demonstrated that effective drowsiness detection can be achieved with band-pass filters operating within the [0.1–30] Hz range. We, therefore, applied a band pass FIR filter with the same frequency range to clean up the EEG signals [37,38,39].

The segmentation phase of EEG signals plays a crucial role in the accuracy of drowsiness detection systems [40]. The segment length must be carefully chosen to balance temporal resolution with the ability to capture meaningful physiological patterns associated with the transition from alertness to drowsiness. Since the shift between wakefulness and drowsiness is typically gradual rather than abrupt, researchers commonly use 30-s epochs, as this duration effectively captures the evolving brain activity during the transition phase. A segment that is too short may fail to reflect these slow physiological changes, while longer segments risk reducing temporal responsiveness and increasing computational demands. Prior studies have demonstrated that 30-s segments provide an optimal trade-off between information richness and detection reliability [41]. Accordingly, in this study, the filtered EEG signals were segmented into 30-s windows to ensure accurate and efficient analysis [42].

### 3.3. Continuous Wavelet Transform

The time-frequency domain [43] offers an integrated approach for analyzing EEG signals in drowsiness detection by combining the temporal and spectral characteristics of the data. Unlike separate time-domain or frequency-domain analyses, it provides a comprehensive exploration of signal variations. The CWT [44], a robust time-frequency analysis method, represents a signal (t) as contributions at different time and frequency scales. The CWT is defined as Equation (1):

(1) $$W\left(l,n\right)=\int_{-\infty }^{\infty }x\left(t\right)\frac{1}{\sqrt{\left|l\right|}}\psi^{*}\left(\frac{t-n}{l}\right)dt$$

where W(l,n) represents the wavelet coefficients, *l* controls the scale (frequency), *n* defines the temporal position relative to time t, and $\psi^{*}$ is the complex conjugate of the mother wavelet ψ(t). This approach enables a multi-resolution analysis, critical for detecting rapid transitions between wakefulness and drowsiness [45]. The resulting scalograms [46], representing graphically the intensity of the transform, facilitate the visualization of frequency variations associated with vigilance states.

**Figure 6:**
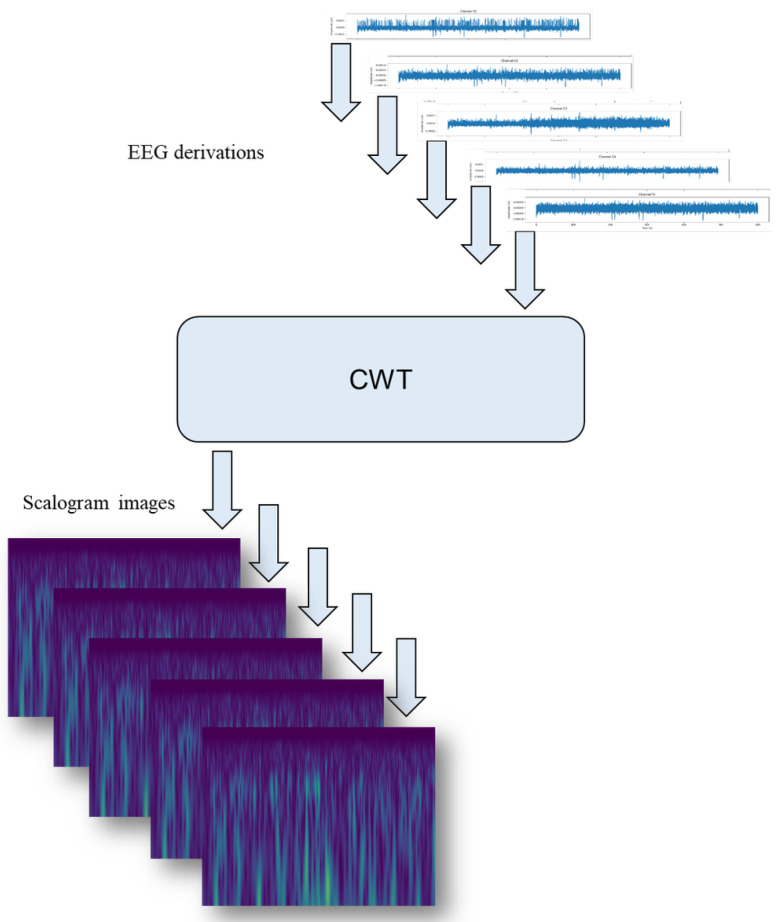
Scalogram generation with continuous Morlet wavelet.

Compared to the Short-Time Fourier Transform (STFT) [47], which uses fixed-size windows and uniform time-frequency resolution, the Wavelet Transform (WT) employs scalable mother wavelets, providing long windows for low frequencies and short windows for high frequencies. This adaptability makes CWT particularly effective for non-stationary EEG signals, allowing the precise localization of events in both the time and frequency domains. In this study, the Morlet wavelet [48] is applied to generate scalogram images, as illustrated in Figure 6 for alert and drowsy subjects. A total of 1400 EEG scalogram images were generated across the five channels for all 14 subjects.

### 3.4. Image Resizing

The scalogram images generated by the CWT applied to EEG data initially have a resolution of 662 × 536 pixels. To adapt these images for analysis with a CNN, they are resized to 256 × 256 pixels using cubic interpolation [49].

This resizing choice was determined after experimenting with various dimensions, with 256 × 256 offering an optimum balance between accuracy and computational efficiency. First of all, CNNs need uniform input dimensions for consistent processing, and resizing to 256 × 256 pixels guarantees this uniformity. Moreover, this resolution optimizes computational complexity, enabling efficient model training while maintaining a reasonable demand on resources. Cubic interpolation is used to effectively preserve essential scalogram details and features, ensuring high-quality input data. Lastly, the resized dimensions facilitate data normalization and significantly improve the convergence and overall performance of the CNN, demonstrating its suitability for this application.

### 3.5. CNN

Convolutional layers [50] extract image features by applying filters (kernels) that detect patterns like edges, shapes, and textures through convolution operations. Each filter generates a feature map, highlighting specific image attributes. Key parameters include filter size (commonly 3 × 3 or 5 × 5 pixels) and the number of filters, which determine the diversity and richness of extracted features. Networks typically use dozens, if not hundreds, of filters per layer, enabling both simple and complex patterns to be efficiently detected.

In this work, we compare a basic 2D CNN with a 1D CNN to highlight the effectiveness of the proposed approach. Additionally, the proposed method is benchmarked against the most commonly used transfer learning models in drowsiness detection, namely, VGG16 and ResNet50, to validate its performance and reliability.

The proposed CNN architecture for feature extraction is implemented using the Keras library, designed with a focus on simplicity and efficiency. The model begins with a convolutional layer [47] comprising 16 filters, a kernel size of (3, 3), and a ReLU activation function [51], applied to input images with dimensions of (64, 64, 3). This layer is followed by a MaxPooling2D operation with a pool size of (2, 2) [52], which reduces the spatial dimensions, and a Dropout layer with a rate of 0.25 to prevent overfitting by randomly deactivating 25% of the neurons during training [53]. The second convolutional layer expands the feature extraction capacity, utilizing 64 filters with the same kernel size and activation function as the first layer. It is similarly followed by a MaxPooling2D layer with a pool size of (2, 2) and another Dropout layer with a 0.25 dropout rate. The extracted feature maps from the convolutional layers are then flattened into a one-dimensional vector using a Flatten layer [54], which serves as input to a fully connected dense layer containing 128 neurons [55] and a ReLU activation function. To further improve generalization, a second Dropout layer with a rate of 0.5 is applied, deactivating 50% of the neurons.

#### 3.5.1. General Hyperparameters

Before detailing each model’s architecture, we first summarize the common hyperparameters applied to all implemented methods. These shared settings ensured a fair training procedure and a consistent evaluation across different models, as shown in Table 1.

#### 3.5.2. Model-Specific Hyperparameters

In addition to the general training settings, each architecture required specific configurations aligned with its design. These details ensured optimal performance and a fair comparison between generic models (i.e., VGG16, ResNet50) and EEG-oriented architectures (i.e., DeepConvNet, EEGNet, ShallowConvNet), as shown in Table 2.

### 3.6. Classification

For the classification phase, we use support vector machines because of their high performance in binary classification and their ability to handle non-linearity.

SVMs are a widely used supervised learning method for classification [56]. They work by identifying the optimal hyperplane that maximizes the separation between classes in the feature space. The equation for the hyperplane can be expressed as shown in Equation (2):

(2) ω·x+b=0

The goal of the SVM is to maximize the margin between the classes. Mathematically, this is translated into minimizing Equation (3):

(3) $$\frac{1}{2}{∥\omega ∥}^{2}$$

subject to the constraint that (4)

(4) $$y_{i}\left(\omega \cdot x_{i}+b\right)\geq 1$$

for all training data ${\left\{\left(x_{i},y_{i}\right)\right\}}_{i=1}^{N}$, where $y_{i}$ represents the class labels.

To handle non-linear data, SVMs use kernel functions such as the linear, polynomial, Gaussian, and Radial basis function (RBF) kernels [57]. These kernels map the data into a higher-dimensional feature space where linear separation becomes feasible. In this context, the optimization problem is formulated as minimizing Equation (5):

(5) $$\frac{1}{2}{∥\omega ∥}^{2}+C\sum_{i=1}^{N}\xi_{i}$$

subject to the constraints in Equation (6):

(6) $$y_{i}\left(\omega^{T}\varphi \left(x_{i}\right)+b\right)\geq 1-\xi_{i},\;\xi_{i}\geq 0$$

where $\varphi (x_{i})$ represents the transformation of the data via the kernel function, and $\xi_{i}$ are the slack variables that allow for margin violations.

The parameter *C* balances the trade-off between maximizing the margin and minimizing classification error. A higher *C* emphasizes minimizing classification errors, while a lower *C* prioritizes a larger margin, potentially allowing some misclassifications.

SVMs are particularly well-suited for the classification of EEG data because they can handle the complexity and non-linearity of EEG signals using appropriate kernels. EEG signals, by nature, exhibit complex and non-linear patterns, making SVMs ideal for this task. By using kernels such as the RBF kernel, SVMs can project EEG data into a feature space where differences between alert and drowsy states become more apparent and linearly separable. This enables SVMs to identify subtle and complex patterns in EEG signals, offering high performance in binary classification.

### 3.7. Performance Evaluation

**Figure 7:**
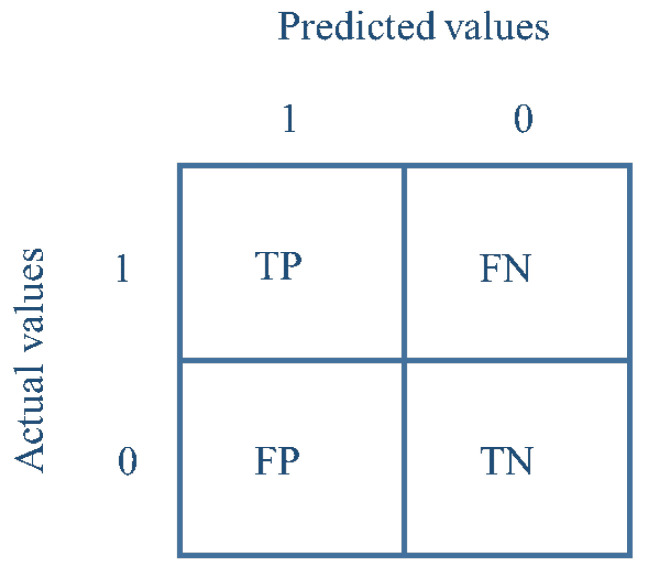
Confusion matrix of binary classification.

To evaluate the classification performance of the detection approach in this work, a binary confusion matrix metric is used [58]. Figure 7 illustrates this confusion matrix. The matrix provides a visual representation of the model’s performance by presenting the number of true positives, false positives, true negatives, and false negatives:

- True positive (TP): prediction of drowsiness when the actual state is drowsiness;
- False positive (FP): prediction of drowsiness when the actual state is alertness;
- True negative (TN): prediction of alertness when the actual state is alertness;
- False negative (FN): prediction of alertness when the actual state is drowsiness.

These elements are essential for calculating various performance measures such as accuracy, precision, specificity, sensitivity, and F-score, thereby offering a comprehensive assessment of the effectiveness of the proposed detection model. The different performance metrics are presented in Table 3.

The DROZY database, as previously mentioned, consists mainly of EEG signals recorded from five electrodes: Fz, Cz, C3, C4, and Pz. In this study, our goal was to develop an optimized approach suitable for integration into an embedded system for drowsiness detection. Based on our recent experiments and previous work, we evaluated the classification accuracy obtained from each individual electrode in the DROZY dataset. We found that the C4 electrode provided the highest accuracy in detecting drowsiness, followed closely by C3 [59]. However, to avoid relying on a single EEG derivation, which could lead to system malfunction in the case of electrode failure or poor contact with the scalp, we decided to test the combination of C3 and C4 electrodes. Our results demonstrated that the use of both electrodes improved classification performance compared to using either one alone [60]. Therefore, we chose to continue with these two electrodes (C3 and C4), as they offer a good trade-off between performance and system simplicity. This selection is also consistent with our objective of designing an approach that is adaptable to the constraints of embedded system implementation, where minimizing the number of electrodes is essential for practicality and user comfort. By combining data from these two electrodes across all subjects, a total of 3360 scalograms were obtained for the wakefulness state and 1680 scalograms for the drowsiness state. To rebalance the dataset before training, a data augmentation technique was applied to the scalograms associated with the drowsiness state. This step resulted in a final balanced dataset composed of 6720 scalograms, evenly distributed between the two classes: 3360 for the wakefulness state and 3360 for the drowsiness state. The objective of this experiment is to evaluate the robustness of the approach with respect to inter-subject variability and to test its generalization capability. To this end, a combined data split was adopted, with 70% used for training and 30% for testing.

## 4. Results and Discussion

In this section, we present the results of the evaluation of drowsiness detection in the inter-subject mode using the proposed approach. This evaluation aims to verify the generalizability of the model by testing its performance on subjects outside the training set. Results are analyzed in terms of performance metrics, such as accuracy, sensitivity, specificity, precision, and F-score. These metrics will allow us to assess the robustness and effectiveness of the approach under inter-subject conditions, where physiological variability is a critical factor. These results will help to validate the approach’s feasibility in practical applications to detect drowsiness in a real-life environment.

### 4.1. Influence of CNN Depth on Drowsiness Detection Accuracy

In this part, we will explore how convolutional depth influences the accuracy of drowsiness detection models. The objective is to assess how varying the number of layers (either increasing or decreasing) affects the model’s ability to extract relevant features from images and, consequently, its overall performance. We will evaluate the results as a function of the number of layers, focusing on the strengths and weaknesses of the different CNN setups. Table 4 presents the performance outcomes for the various CNN configurations, where $CNN_{1}$ consists of one convolutional layer, $CNN_{2}$ has two layers, $CNN_{3}$ includes three layers, $CNN_{5}$ is composed of five convolutional layers, and $CNN_{10}$ features a deeper architecture with 10 convolutional layers.

The results indicated that the $CNN_{3}$ model, which consists of three convolutional layers, demonstrates the highest efficiency in extracting features relevant to drowsiness detection.

### 4.2. Combined CNN-SVM Model

In order to improve the classification of our drowsiness detection system, we apply the SVM technique to the features extracted by the CNN. To determine the optimal SVM kernel, we evaluate multiple options, including linear, polynomial, RBF, and sigmoid kernels. Table 5 summarizes the classification results for each of these SVM kernels.

The results indicate that the RBF kernel was the most effective among those tested (more than 2% improvement). While the linear, polynomial, and sigmoid kernels also performed well, the RBF kernel achieved an impressive accuracy of 98.33%, distinguishing itself through its ability to model non-linear decision boundaries. This is particularly important for detecting drowsiness, where EEG signal relationships tend to be complex and non-linear. Furthermore, the RBF kernel demonstrates superior generalization on test data, reducing the likelihood of overfitting. Its flexibility enables it to pick up subtle variations in the signal, improving the accuracy of drowsiness detection. The SVM-RBF hyperparameters, penalty factor C = 1 and kernel factor γ=0.4, were selected using the GridSearch optimization tool.

**Figure 8:**
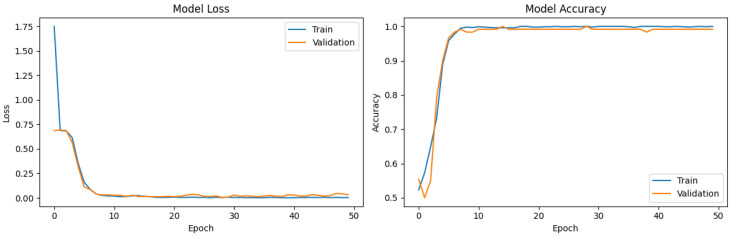
Loss and accuracy curves for training (blue) and test (red) of the proposed using CNN SVM (RBF) method.

**Figure 9:**
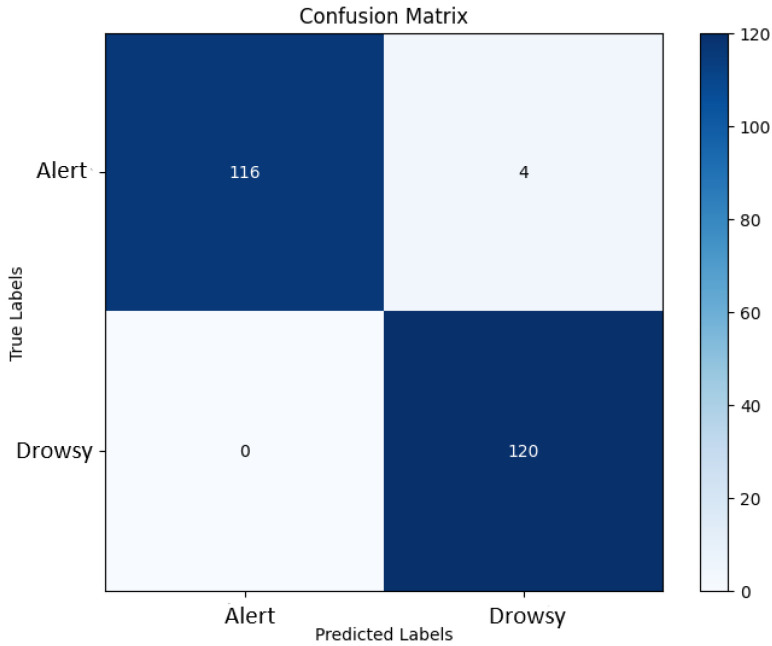
The confusion matrix of the proposed approach.

Moreover, replacing the final softmax layer of the CNN3 model with an SVM classifier led to an increase in accuracy of 0.53%. This improvement can be explained by the intrinsic differences between the two classifiers: While the softmax layer functions as a linear classifier optimized for binary classification through cross-entropy loss, it may struggle with complex, high-dimensional, and non-linearly separable EEG feature spaces. In contrast, the SVM with an RBF kernel is designed to find an optimal separating hyperplane in a transformed feature space, maximizing the margin between the two classes and effectively handling non-linear relationships. This allows the SVM to better capture subtle non-linear EEG patterns associated with drowsiness, especially in the high-dimensional feature space extracted by CNN layers, thus enhancing classification performance. The accuracy and loss curves (during training and test) for the CNN SVM (RBF) are shown in Figure 8. Figure 9 illustrates the confusion matrix for the classification operation.

The evolution of the loss and accuracy curves highlights the efficient convergence of the model. The loss curve shows a rapid decrease during the initial epochs, stabilizing near zero after approximately 10 iterations. The validation loss follows a similar trend, with minor fluctuations in later epochs, suggesting good generalization capabilities.

Regarding accuracy, a sharp increase is observed during the first few epochs, reaching a plateau close to 99% by the tenth iteration. The absence of a significant gap between the training and validation curves indicates that the model neither underfits nor overfits. These results suggest optimal convergence and a strong ability to generalize to unseen data.

The analysis of the confusion matrix reveals high classification performance. The model correctly identified 116 samples from class 0 and 120 samples from class 1. In this context, class 0 refers to the alert state, while class 1 refers to the drowsy state. However, four samples from class 0 were misclassified as class 1, introducing false positives. Notably, no false negatives were observed, indicating perfect sensitivity in detecting class 1.

These results demonstrate high precision and recall, confirming the robustness of the model. The absence of false negatives is particularly beneficial for critical applications where detecting class 1 is essential. Although the presence of a few false positives may lead to unnecessary alerts, their low occurrence suggests that the model maintains reliable and robust classification performance.

**Figure 10:**
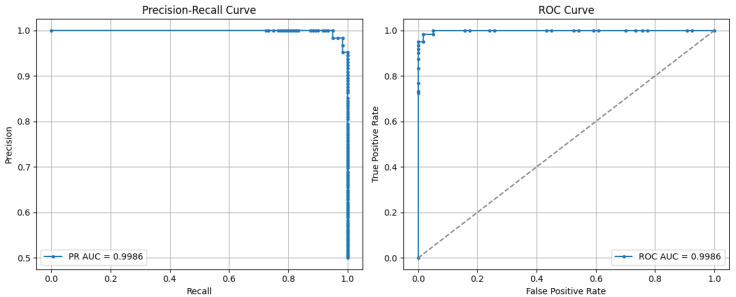
The PR and ROC ACU for the proposed method.

Figure 10 presents two complementary evaluation curves for the model: the precision–recall curve (left) and the ROC curve (right). The precision–recall curve, with an area under the curve (PR AUC) of 0.9986, lies almost entirely in the upper-right region, reflecting very high precision and recall across all decision thresholds. This shape indicates that the model maintains excellent precision even when recall is maximized, which is particularly relevant for potentially imbalanced datasets. The ROC curve, with an area under the curve (ROC AUC) of 0.9986, shows a nearly vertical rise followed by a plateau close to 1, illustrating an almost perfect discriminative ability between positive and negative classes. These results confirm that the model effectively detects the positive class while minimizing false positives, thereby demonstrating outstanding overall performance.

### 4.3. Comparison Between the Proposed Method and 1D CNN

This experiment aims to evaluate the effectiveness of the feature extraction method for drowsiness detection by comparing a 2D CNN + SVM model, which processes time-frequency representations (scalograms), with a 1D CNN that directly analyzes raw EEG signals.

The results in Table 6 demonstrate that the 2D CNN + SVM approach outperforms the 1D CNN, highlighting the advantage of leveraging time-frequency features for capturing relevant patterns in drowsiness detection. This comparison highlights the importance of using advanced feature extraction techniques, such as scalograms, to improve the performance of EEG-based drowsiness detection systems.

The results clearly show that the proposed method (2D CNN + SVM) is much more effective at detecting drowsiness and overcoming inter-subject variability compared to CNN 1D. Specifically, the performance of the proposed approach achieved an accuracy of 98.33%, while CNN 1D reached 72.79%, highlighting a significant improvement of approximately 35.09%.

### 4.4. Comparison with Transfer Learning Models

In this section, we compare the proposed 2D CNN + SVM approach with transfer learning models, specifically, VGG16, ResNet50, DeepConvNet, EEGNet, and ShallowConvNet, to evaluate its effectiveness for drowsiness detection in an inter-subject context. The goal is to demonstrate the advantages of the proposed method, particularly the integration of SVM in the classification phase, which enhances the system’s performance and robustness.

Transfer learning leverages pre-trained models trained on large datasets, enabling them to capture general features that can be fine-tuned for specific tasks like drowsiness detection. Transfer learning models provide strong initial feature extraction, which can be adapted to EEG data characteristics, offering a robust starting point for domain-specific tasks. By contrasting the results with established transfer learning models, we aim to highlight the superior accuracy, sensitivity, and F1-score of the 2D CNN + SVM approach, as illustrated in Table 7. This comparison underscores the importance of tailored feature extraction and classification techniques in improving drowsiness detection systems.

For the comparison of our proposed approach with state-of-the-art models including 1D-CNN, VGG16, ResNet50, DeepConvNet, EEGNet, and ShallowConvNet, we employed commonly accepted standard hyperparameters as reported in the EEG signal processing literature. All models were trained for 50 epochs using the Adam optimizer with learning rates adapted to each architecture to ensure fair and effective convergence. The selection of hyperparameters was conducted through a grid search procedure to optimize model performance systematically.

Specifically, the 1D-CNN was configured with a learning rate of 0.001, a batch size of 32, a dropout rate of 0.5, and kernel sizes of 64 and 32 in its convolutional layers with ReLU activations. VGG16 and ResNet50, pretrained on ImageNet, were fine-tuned with a learning rate of 1 × 10^−4^, batch size of 32, and dropout of 0.5 applied on fully connected layers; these models use 3 × 3 kernels for VGG16 and a combination of 7 × 7 initial kernels followed by bottleneck blocks for ResNet50, all with ReLU activations. The EEG-specific architectures DeepConvNet, EEGNet, and ShallowConvNet were trained with a learning rate of 0.001, batch size of 64, and dropout rates ranging from 0.25 to 0.5. DeepConvNet and EEGNet utilize ELU activation functions, while ShallowConvNet applies square and logarithmic non-linearities suited for oscillatory EEG features. Kernel sizes varied per model according to their original designs: DeepConvNet uses temporal and spatial kernels of size [1 × 10], EEGNet employs depthwise and separable convolutions with kernels of sizes [1 × 64] and [1 × 16], and ShallowConvNet uses a kernel size of [1 × 13]. Batch normalization was applied after convolution layers in all models to stabilize training. This standardized configuration ensured consistent and fair evaluation of all models on the same datasets, allowing for a rigorous comparison of performance.

### 4.5. Discussion

The findings of this study highlight the potential of the proposed EEG-based method for drowsiness detection, achieving notable accuracy even when applied across different subjects. However, while the CWT successfully generated scalogram images that captured detailed time-frequency features, the method’s real-world practicality remains questionable. The reliance on CWT to extract meaningful information, although beneficial in revealing the evolution of frequency content over time, may introduce complexity and computational overhead. Furthermore, while this approach is an improvement on traditional methods of representing non-stationary signals such as EEG, its scalability and performance in more varied and less controlled environments still need to be proven, leaving room for further refinement and validation. Although the use of CNNs for automatic feature extraction has shown great promise for detecting patterns associated with drowsiness, it is essential to critically evaluate the wider implications of this approach, particularly in terms of its impact on detection accuracy and system performance. The implementation of an SVM allows efficient differentiation between drowsy and alert states in a cross-subjects framework, improving the robustness of the model to individual variability. However, it is worth asking whether this combination of CWT, CNN, and SVM effectively offers a superior solution to existing methods. Although the preliminary results are encouraging, further validation with diverse populations and under real-world conditions is needed to confirm that it systematically outperforms previous approaches in real-world applications.

In Table 8, we compare our proposed method with existing literature on drowsiness detection in an inter-subject setting. In [23], the authors employed a simple 1D CNN for drowsiness detection using 10-s EEG segments as input for classification. The final dense softmax layer of the CNN was responsible for decision-making, achieving an accuracy of 73.22%. In [24], the same approach was followed, except for a reduction in EEG segment length to 4 seconds. This modification resulted in a significant accuracy improvement to 95%, highlighting the crucial impact of segment length on detection performance.

In another study [25], the authors utilized EEG spectrograms for drowsiness detection, employing an RCNN model that achieved an accuracy of 88.39%. Similarly, in [26], a CNN-LSTM architecture was used to predict drowsiness states based on EEG spectrograms, reaching an accuracy of 82.73%. In [27], EEG epochs of 13 seconds were used as input features for a CNN-based model (CpNet), which achieved an accuracy of 86.44%. Finally, in [28], 5-s EEG epochs were directly fed into a CNN, resulting in an accuracy of 93%. In this work [61], the authors propose a deep learning model called AMD-GCN, which utilizes the power spectral density (PSD) of EEG signals filtered with a band-pass filter ([1–50] Hz) from 17 electrodes for drowsiness detection. The model was validated on the SEED-VIG database and achieved an overall accuracy of 89.94%. While this approach demonstrates strong performance in the field of drowsiness detection, it remains limited in terms of implementation on embedded systems due to its algorithmic complexity. Furthermore, the authors did not address inter-subject variability, as the evaluation was performed only in the intra-subject mode with a relatively high number of required electrodes.

In the work [62], the authors proposed a drowsiness detection approach focusing on minimizing inter-subject variability. In this context, they employed a Random Forest (RF) classifier, achieving an accuracy of 86%. The method was validated using the SEED-VIG database, extracting power spectral density (PSD) features from 8-s EEG epochs. While this approach is optimized from an implementation perspective, its accuracy remains relatively low, particularly in the context of subject-independent detection. Furthermore, the method focuses exclusively on a single type of feature (PSD), which may limit its generalization capability.

**Table 8:** Comparison of different EEG-based drowsiness detection methods.

Ref	Method	Segment Size	Classifier	Database	A (%)
[24]	1D EEG + CNN	4 s	Fully connected layer with Softmax activation	SEED-VIG	95.00
[25]	Spectrogram + RCNN	–	Fully connected layer with Softmax activation	SEED-VIG	88.39
[26]	Spectrogram + CNN-LSTM	–	Fully connected layer with Softmax activation	SEED-VIG	82.73
[63]	AGL-Net	–	Fully connected layer with Softmax activation	SEED-VIG	87.30
[64]	VIG-Net	8 s	Fully connected layer with Softmax activation	SEED-VIG	95.00
[61]	AMD-GCN model	10 s	Fully connected layer with Softmax activation	SEED-VIG	89.94
[62]	ML+PSD	8 s	RF	SEED-VIG	86
Proposed	2D CNN + SVM	30s	SVM	SEED-VIG	95.8

**Note:** A is accuracy.

The literature analysis clearly demonstrates that the proposed method outperforms existing approaches, achieving an accuracy of 98.33%. This result indicates that our approach effectively mitigates inter-subject variability due to several key factors. One crucial factor is the length of EEG epochs, which plays a significant role in drowsiness detection. The use of 30-s epochs provides a more comprehensive representation of vigilance states compared to shorter segments of 1 s, 4 s, 5 s, or 13 s.

Another major contributor to the improved accuracy is the use of scalograms, which offer a time-frequency representation of EEG signals derived from the Continuous Wavelet Transform (CWT). Unlike raw EEG signals, which are challenging to interpret due to their non-stationary nature, or spectrograms generated via the Fast Fourier Transform (FFT), which provide only a frequency-domain representation, scalograms capture both temporal and spectral information. Additionally, CNNs are highly effective in processing images, making scalogram-based feature extraction particularly relevant. CNNs can automatically learn meaningful features associated with drowsiness, further enhancing classification performance.

Finally, the choice of a classification model that excels in binary classification, while effectively handling non-linearity and high-dimensional data, significantly improves detection accuracy compared to a simple dense softmax layer. Through the analysis of previous studies, we observe that one of the most widely used datasets for drowsiness detection research is the SEED-VIG database.

To assess the ability of our approach to minimize inter-subject variability and ensure robust generalization, we evaluated its performance on the SEED-VIG database. This dataset contains EEG recordings from 21 subjects, collected using 12 electrodes (CP1, CPz, CP2, P1, Pz, P2, PO3, POz, PO4, O1, Oz, and O2). Each participant underwent a two-hour driving simulation during two key periods of the day—the afternoon and the evening—when drowsiness levels are most likely to vary. From these recordings, 5040 scalograms were generated for analysis. The results show a slight performance degradation compared to the original DROZY dataset, with an accuracy of 95.8%, a sensitivity of 94.7%, and an F1-score of 95.4%, against 98.33% accuracy on DROZY. This performance drop can be primarily attributed to the difference in electrode configurations between the two datasets, particularly the absence of the C3 and C4 derivations in SEED-VIG. These central electrodes are known to capture cortical activity highly relevant to vigilance regulation and early drowsiness onset, and their absence reduces the discriminative information available to the classifier.

To further investigate the robustness and generalizability of our approach across different experimental conditions and subject populations, an additional validation was conducted using an independent dataset collected at the Sahloul University Hospital (Monastir, Tunisia). This dataset comprises 45 hours of EEG recordings from eight healthy subjects aged between 21 and 25 years, all without a history of alcoholism or drug use. Data collection was performed at the Vigilance and Sleep Center of the Faculty of Medicine in Monastir, following an experimental protocol approved by the faculty’s Ethics Committee. All participants signed an informed consent form prior to the experiment, in compliance with ethical research standards. EEG signals were recorded from 19 channels (Fp1, Fp2, F2, F3, Fz, F4, F8, T3, C3, Cz, C4, T4, T5, P3, Pz, P4, T6, O1, and O2).

For validation purposes, only the C3 and C4 electrodes were retained, ensuring consistency with our minimal-electrode configuration. On this dataset, the proposed method achieved an accuracy of 97.83%, which is only marginally lower than the performance obtained on the DROZY database. These results demonstrate that the approach is capable of maintaining high detection performance even when applied to EEG data collected from different populations, under varying recording setups, and in diverse environments. The ability to achieve comparable performance with minimal electrodes across heterogeneous datasets strongly supports our claim that the proposed method effectively mitigates inter-subject variability and remains suitable for practical drowsiness monitoring applications, where electrode configurations and acquisition protocols may differ from one scenario to another. To assess whether the observed differences in performance across datasets were statistically significant, we conducted independent *t*-tests with corresponding *p*-value analysis. The comparison between the DROZY (98.33%) and SEED-VIG (95.8%) datasets revealed a highly significant difference (t = 23.72, *p* < 0.001), confirming that the absence of C3 and C4 electrodes in SEED-VIG had a measurable impact on performance. A smaller but significant difference was also found between DROZY (98.33%) and the Sahloul dataset (97.83%) (t = 5.56, *p* < 0.001). Finally, SEED-VIG and Sahloul also showed a statistically significant difference (t = −18.33, *p* < 0.001), highlighting the role of electrode configurations and acquisition protocols in shaping classification outcomes. Despite these statistical differences, all datasets consistently achieved accuracies above 95%, demonstrating that the proposed method remains robust and generalizable across heterogeneous populations and experimental conditions.

## 5. Conclusions

Inter-subject variability represents a major challenge in EEG-based drowsiness detection, often limiting the reliability and generalizability of existing systems. In this study, we addressed this issue by proposing a hybrid detection approach that leverages the Continuous Wavelet Transform (CWT) with the Morlet wavelet to generate scalogram images, thereby capturing both temporal and spectral information associated with vigilance states. These scalograms were resized and processed by a lightweight CNN, specifically designed to maintain computational efficiency while ensuring high discriminative power. The extracted features were then classified using an SVM with an RBF kernel, which excels in handling the non-linear and high-dimensional nature of EEG data. This combination achieved an accuracy of 98.33% in inter-subject scenarios, confirming its ability to mitigate variability between subjects and provide a highly generalized and accurate detection system.

The novelty of the approach lies not only in the CNN–SVM fusion, but also in its integration of several complementary strategies: (i) the use of 30-s epochs to capture gradual vigilance transitions, (ii) reliance on a minimal-electrode configuration (C3 and C4) to enhance practicality for real-world use, (iii) feature standardization to further reduce inter-subject variability, and (iv) the adoption of a lightweight CNN architecture optimized for FPGA-friendly implementation. These design choices collectively ensure a strong balance between accuracy, robustness, and computational efficiency, with results consistently above 95% across heterogeneous datasets.

Looking ahead, we plan to extend this work in two directions. First, the integration of complementary physiological signals (i.e., EOG, ECG) and facial expressions will be explored to further improve robustness. Second, as suggested by recent advances, we acknowledge the potential of super-resolution techniques for time–frequency mapping and more advanced classifiers such as Vision Transformers (ViT) and large vision models like CLIP. While these models currently demand high computational resources and are less suited to embedded contexts, future investigations will explore lightweight adaptations of such architectures. This direction aims to combine the strength of transformer-based models with our embedded implementation strategy, ensuring that drowsiness detection systems remain both accurate and deployable in real-time portable platforms such as FPGAs.

## Figures and Tables

**Table 1:** General hyperparameters used across all models.

Hyperparameter	Configuration
Optimizer	Adam
Learning rate	Adapted per architecture (see Table 2)
Loss function	Binary cross-entropy
Epochs	50
Batch size	32 (VGG16, ResNet50, 1D-CNN), 64 (DeepConvNet, EEGNet, ShallowConvNet)
Evaluation metrics	Accuracy, Sensitivity, Specificity, F1-score, AUC
Early stopping	Enabled, patience = 10 epochs
Batch normalization	Applied after each convolutional layer
Hyperparameter optimization	Grid search procedure

**Table 2:** Architecture-specific hyperparameters for each model.

Model	Configuration
1D-CNN	LR = 0.001, batch size = 32, dropout = 0.5, ReLU activation, kernel sizes [64, 32]
VGG16 (fine-tuned)	Pretrained on ImageNet, LR = 1 × 10^−4^, batch size = 32, dropout = 0.5 on FC layers, 3 × 3 kernels, ReLU activation, frozen lower layers
ResNet50 (fine-tuned)	Pretrained on ImageNet, LR = 1 × 10^−4^, batch size = 32, dropout = 0.5 on FC layers, initial 7 × 7 convolution + bottleneck blocks, ReLU activation, frozen lower layers
DeepConvNet	LR = 0.001, batch size = 64, dropout = 0.5, ELU activation, temporal and spatial kernels [1 × 10]
EEGNet	LR = 0.001, batch size = 64, dropout = 0.25, ELU activation, depthwise and separable convolutions [1 × 64], [1 × 16]
ShallowConvNet	LR = 0.001, batch size = 64, dropout = 0.5, square and log activations, kernel size [1 × 13]

**Table 3:** Mathematical formulations of performance metrics.

Metric	Formula
Accuracy	$\frac{TP+TN}{TP+FP+FN+TN}$
Sensitivity (Recall)	$\frac{TP}{TP+FN}$
Specificity	$\frac{TN}{TN+FP}$
Precision	$\frac{TP}{TP+FP}$
F1-score	$2\times \frac{\mathrm{Precision}\times \mathrm{Recall}}{\mathrm{Precision}+\mathrm{Recall}}$

**Table 4:** Performance metrics for each CNN model.

Metric	${\mathrm{CNN}}_{3}$	${\mathrm{CNN}}_{10}$	${\mathrm{CNN}}_{2}$	${\mathrm{CNN}}_{5}$	${\mathrm{CNN}}_{1}$
Accuracy (%)	97.9	94.6	93.5	92.7	88.78
Sensitivity (%)	96.73	94.4	94.2	93.5	87.9
F1-score (%)	96.92	95.3	93.2	93.7	88.5
Precision (%)	97.5	95.1	93.8	93.9	89.2
Specificity (%)	98.1	94.9	94.5	92.8	88.3

**Table 5:** Performance metrics for each SVM kernel.

Kernels	A (%)	S (%)	F1 (%)	Sp (%)	P (%)
SVM-RBF	98.33	100	98.36	96.67	96.77
SVM-Linear	95.21	97.95	95.5	93.7	93.79
SVM-Polynomial	85.2	86	85.3	84.7	84.5
SVM-Sigmoid	70.85	70.2	70.98	69.7	70.1

A: accuracy, S: sensitivity, F1: F1-score, Sp: specificity, P: precision.

**Table 6:** Superior performance of 2D CNN + SVM in drowsiness detection over 1D CNN. A: accuracy, S: sensitivity, F1: F1-score, Sp: specificity, P: precision.

Model	A (%)	S (%)	F1 (%)	Sp (%)	P (%)
2D CNN + SVM	98.33	100	98.36	96.67	96.77
CNN 1D	72.78	72.89	72.20	71.90	72.30

**Table 7:** Comparison between the proposed approach and transfer learning models.

Model	A (%)	S (%)	F1 (%)	Sp (%)	P (%)
2D CNN +SVM	98.33	100	98.36	96.67	96.77
VGG 16	52.55	57.80	53.20	51.30	52.70
ResNet 50	87.50	88.30	87.70	86.40	87.20
DeepConvNet	88.72	89.03	88.05	88.45	87.12
EEGNet	87.56	88.10	87.44	87.30	86.80
ShallowConvNet	88.86	89.15	88.67	88.60	88.2

## Data Availability

The original data presented in the study are openly available DOI:10.1109/WACV.2016.7477715.
